# Over-eating

**Published:** 1882-01

**Authors:** 


**OVER-EATING.**—"I am the captive of appetite. I am hungry all the time, and get up from the table hungry." Thus writes a public man of unusual promise. A great name once said: "I have been hungry for two years." He had a malady which threatened death, if he ever over-ate, yet he had the force of will to avoid excesses for a lifetime. His name and his works will live in the memory of the good for ages to come. To avoid over-eating requires moral courage; it takes a man to do it; cowards and babies fail every day. An incessant feeling of hunger is a great torment; it is the sign of dyspepsia. A dyspeptic lives on thorns. If he does not satisfy his appetite, there is a ceaseless longing to eat; if he does eat as much as he wants, he either spits it up by piece-meal in the course of hours, or suffers a variety of aches and ails which make of life a burden, and utterly unfit him for enjoyment, or the proper discharge of business or of duty. The very first step towards the cure of any case of dyspepsia, is the heroic resistance to the calls of the stomach. The rule, in almost all cases, is to eat but little, eat often, eat regularly, and to be in active exercise in the open air for as many hours of each day as possible; the more the better. The very essence of dyspepsia is, that the stomach is too weak to manage the food introduced into it; it can not convert it into nutriment. The work of the stomach has been compared to a kind of churning operation; it carries the food round and round, as a spoon carries bits of ice in a glass of water, when they are to be melted. Now if the stomach is too weak to push them around, they settle, remain at rest, collect at one point, and we speak of it as a "load," as being "heavy as lead." To carry out the comparison so as to convey the desired idea, at the expense of a literal scientific view of the case, the stomach might be able to carry a little food, an ounce around its walls, when it could not carry a full meal of two pounds; it may carry around a few ounces of plain, nourishing food, and digest it, dissolve it in an hour or two, when it would fail to do the same in five hours, by a hearty meal. A faithful servant recovering from a long sickness may be able to do a little work, and do it properly; but if too much is given, it is either not done at all, or not done well. By giving a little at a time, and giving opportunity for rest, an ability is gradually acquired to do more and more.

The general rules for dyspeptics are, eat what you crave, but only so much as will not afterwards give the slightest discomfort whatever, and gradually feel the way along to take more and more. The easiest way to avoid eating too much, is to have, at specified times, that amount of food sent to your room, which observation has shown to be proper for you. If this plan is persevered in, the feeling of gnawing hunger will gradually disappear, and will only be present when the time for eating comes. Generally speaking, in our own observation, the best bread for a dyspeptic is pilot bread, or ship-biscuit, having nothing in them but flour and water, or the crust of cold wheat or light bread, in either case softened with hot water; fresh meat, rare done, and cut up as fine as a pea, is more easily digested and converted into nutriment and strength than any vegetable whatever. Ship-biscuit, fresh meat, and abun dant out-door work will cure almost any dyspeptic, if the use of these is persevered in according to the principles laid down. But as not one in ten thousand has the moral courage to practice the self-denial, and to do the work suggested, this article perhaps might as well have not been written, except that now and then a hero and a philosopher might see it.

